# Effects of the fermentation quality and microbial community of waxy maize mixed with fodder soybean silage

**DOI:** 10.3389/fmicb.2024.1405018

**Published:** 2024-05-03

**Authors:** Mei Yang, Fengdan Wang, Wen Xu, Xiaoming Li, Hang Yin, Muzhapaer Tuluhong, Rui Qiu, Bing Li, Guowen Cui

**Affiliations:** Department of Grassland Science, College of Animal Science and Technology, Northeast Agricultural University, Harbin, China

**Keywords:** waxy maize, fodder soybean, mixed sowing ratio, silage quality, microbial community

## Abstract

Waxy maize (*Zea mays* L. sinensis Kulesh) is highly regarded for its high nutritional content and unique taste. Although the stalks and leaves contain high carbohydrate levels after ear harvesting, inadequate crude protein (CP) limits the utilization and promotion of waxy maize silage in animal husbandry. In this study, waxy maize and fodder soybeans were mixed for sowing in different proportions [1:0 (CK), 1:1 (A1), 1:2 (A2), 1:3 (A3), and 1:4 (A4)] to investigate the effects of different mixing ratios on the growth of the waxy maize, the chemical indices, fermentation quality, and the microbial community of the mixed silage after ear harvesting. The mixed planting of waxy maize and fodder soybeans in different proportions had no effect on the yield and quality of the waxy maize ears and increased the aboveground biomass after ear harvesting. After ear harvesting, the neutral detergent fiber (NDF) and acid detergent fiber (ADF) contents significantly decreased, and the CP content and relative feeding value (RFV) gradually increased in the mixed silage. The pH of the treatments was lower than 4.2 except for A4, and the lowest ammonia nitrogen (AN) concentration was observed in A3. With increasing proportions of fodder soybeans, the abundance of beneficial bacteria increased and that of harmful bacteria decreased; *Firmicutes* and *Lactobacillus* were the dominant phylum and genus, respectively, and both increased gradually. Redundancy analysis (RDA) revealed that the fermentation indices affecting the microbial community composition in the silage were inconsistent among the different mixed sowing combinations. The Mantel test showed that the composition of the microbial communities in the treatments was significantly correlated with the ADF, water-soluble carbohydrate (WSC), and propionic acid (PA) contents. Comprehensive analysis revealed that the optimal mixed sowing ratio of waxy maize to fodder soybeans was 1:3, and waxy maize and fodder soybeans silage can increase the utilization of aboveground biomass and improve the fermentation quality and feeding quality of silage by changing the microbial community. These findings lay a certain theoretical foundation for improving the utilization of waxy maize.

## 1 Introduction

Waxy maize (*Zea mays* L. sinensis Kulesh), also known as sticky maize, is the earliest cultivated maize found in China (Collins, 1920). As an important food crop and commercial crop, waxy maize is rich in nutrients and has a unique taste due to its high starch content, water-soluble polysaccharides, and multiple vitamins; the fresh fruit spikes or corn grains are harvested during the milk and dough stages of growth (Ruanjaichon et al., 2022). In addition, waxy maize can be used in papermaking, textiles, medicine, etc.; thus, its economic value is much greater than that of ordinary seed maize (Bao et al., 2012; Zheng et al., 2013). After maize ear harvesting, the sugar content and other nutrients in waxy maize straw are much greater than those in ordinary maize straw, which is a high-quality raw material for making silage. However, since the waxy maize ear accounts for 60–70% of the whole plant, the protein content of waxy maize straw decreases after ear harvesting.

Mixed sowing is a forage planting method in which two or more kinds of herbage are sown together in the same soil. As the most common planting method for artificial grasslands, the mixed sowing of gramineous and leguminous forages can lead to full utilization of the complementary advantages of ecological niches, increase the efficiency of resource utilization, and effectively increase grassland biomass; this method effectively combines the protein of leguminous forages with the carbohydrates of gramineous forages to improve forage nutritional quality (Diba and Geleti, 2013; Aponte et al., 2019). In addition, gramineous grasses can also change the nitrogen fixation capacity of leguminous grasses (Dou et al., 2022). The mixed sowing of *Elymus nutans*, *Poa pratensis,* and *Festuca sinensis* increased not only the soil’s total nitrogen and water content, but also the aboveground and belowground biomass (Zhao et al., 2024). The different proportions and modes of mixed sowing not only affect the forage yield (Oruh and Tan, 2016; Guretzky and Redfearn, 2021), but also change the maximum land equivalent ratio, competitive ratio, and actual yield loss (Chaechian et al., 2022).

Fodder soybean (*Glycine max*), widely cultivated in North China, is a high-quality leguminous forage crop because of its high yield, high protein content, and strong shading resistance (Dadson et al., 2015). Silage is a preservation technique that involves the conversion of sugar into lactic acid (LA) by microbial fermentation under anaerobic conditions, which ultimately inhibits the proliferation of undesirable bacteria so that green feed can be preserved for a long time (Fabiszewska et al., 2019). The application of silage not only effectively maintains the fresh green state of forage but also reduces nutrient loss; it can also alleviate the shortage of green feed in Northeast China in winter (Gordon and Steen, 2000). Mixed silage is a storage method that prolongs feed storage time, regulates water and sugar content, improves the raw material utilization rate, adjusts the feed supply period, and improves forage digestibility and palatability for animals. Mixed silage of legume forages such as alfalfa and red clover and grass forages such as ryegrass can improve silage fermentation characteristics (Jatkauskas et al., 2015). The mixed silage of *Festuca elata*, *Avena sativa* straw, and *Medicago sativa* has good fermentation quality and significantly greater nutritional value and *in vitro* digestibility, and it has been reported that the best effect was achieved when the proportion of alfalfa was 30% (Wang et al., 2018). High-quality silage can be obtained by mixing whole maize with alfalfa; intercropping alfalfa with maize can improve the digestibility of mixed silage; and the ratio of alfalfa to maize can be used to regulate digestibility and fermentability (Zhang et al., 2017).

The mixed sowing of waxy maize and fodder soybeans not only maximizes the semi-intertwined properties of fodder soybeans but can also improve the nutritional value of the mixed forage after silage. However, relatively few studies have investigated the mixed sowing and silage of waxy maize and fodder soybeans. Therefore, in this study, we analyzed waxy maize ear yield and quality, aboveground biomass, mixed silage quality, and the microbial community under different mixed sowing ratios of waxy maize and fodder soybean to determine the best mixed sowing ratio for increasing planting income and providing high-quality silage for animal husbandry production. It is important to make full use of land and space resources, reduce pollution caused by straw burning, and ensure the health of the ecological environment.

## 2 Materials and methods

### 2.1 Description of site

The experimental site was located at the experimental base of Northeast Agricultural University, Xiangfang District, Harbin, Heilongjiang Province (N45°75′, E126°73′). The region has a semihumid and semiarid temperate continental monsoon climate, in which the average annual precipitation is 530 mm, the average sunshine duration is 2,786 h, the average annual temperature is 3.6°C, and the average frost-free period is approximately 150 days. This area is flat in the west and hilly and low-lying in the east and southeast, where the soil layer is mostly composed of clay and sand. The soil pH is 6.2 and includes organic matter (28.4 g/kg), total nitrogen (1.5 g/kg), available phosphorus (66.7 mg/kg), and available potassium (112.5 mg/kg).

### 2.2 The experimental design

Waxy maize 626 (*Z. mays*) seeds were obtained from the Maize Research Institute of the Heilongjiang Academy of Agricultural Sciences, and fodder soybean (*G. max*) seeds were obtained from the Grass Laboratory of Northeast Agricultural University. The experimental design was as follows: waxy maize monoculture (CK), one waxy maize with one fodder soybean (A1), one waxy maize with two fodder soybeans (A2), one waxy maize with three fodder soybeans (A3), and one waxy maize with four fodder soybeans (A4), with a row spacing of 65 cm, and a plant spacing of 33 cm. A total of 15 plots (the CK and four mixed sowing treatments with three replicates) were fertilized with 600 kg/hm^2^ of compound fertilizer (N:P_2_O_5_:K_2_O = 26:12:12) at a depth of 10 cm-15 cm.

### 2.3 Analysis of chemical composition and fermentation quality

At the end of the milk stage, the weights of the waxy maize ears, waxy maize straw, and aboveground biomass (the total weight of waxy maize straw and fodder soybeans) were measured. At this point, the fodder soybeans had reached about their full bloom stage. The length of the ears of the waxy maize plants was greater than 16.0 cm, and the length of the first-grade commercial ears ranged from 20.0 to 21.9 cm (Ding et al., 2022). The percentage of the first-grade commercial ears was calculated according to the following formula. The percentage of first-grade commercial ears (%) = the number of first-grade commercial ears/the number of ears × 100%.

The waxy maize straw/fodder soybean plants were chopped to 1.5–2.0 cm and placed into silage vacuum bags (35 cm × 50 cm). The mixed silages were processed with a vacuum packaging machine (Songben Machinery Co, Ltd., Qingdao, China) to extract excess air for filling and compaction. There were five treatments with three replicates per treatment, and the quality of the silage was determined after 45 days of ensiling.

Sensory evaluation of the silage samples was performed (Li et al., 2024), and electronic nose analysis was performed using an electronic nose (DM6, INSENT, Japan) (Cervellieri et al., 2022). The dry matter (DM) content was determined by drying the silage samples to a constant weight in an oven at 65°C (Wang et al., 2022). The dried samples were pulverized and passed through a 40-mesh screen for subsequent measurements. The crude protein (CP) content was determined using the Kjeldahl method (Kang et al., 2023). The crude fat (EE) content was determined using the Soxhlet extraction method (Sosnowski and Truba, 2023). The acid detergent fiber (ADF) and neutral detergent fiber (NDF) contents were determined using the normal detergent fiber method (Van Soest et al., 1991). The soluble carbohydrate (WSC) content was determined using the anthrone colorimetric method (McDonald and Henderson, 1964). The crude ash (Ash) content was determined using the high-temperature burning method in a muffle furnace (Sosnowski and Truba, 2023). The relative feeding value (RFV) was calculated according to the method described by Kang et al. (2023). The samples (20 g) were mixed and homogenized with sterile water (180 mL) and filtered through four layers of gauze for the determination of fermentation indicators. The pH was measured with a glass electrode pH meter (Sartorius Basic pH Meter, Gottingen, Germany) (Ren et al., 2015). The concentration of ammonia nitrogen (AN) was calculated by the phenol-sodium hypochlorite colorimetric method (Wang et al., 2022). High-performance liquid chromatography was used to determine the contents of organic acids, including lactic acid (LA), acetic acid (AA), propionic acid (PA), and butyric acid (BA) (Yuan et al., 2015).

### 2.4 Silage sample DNA extraction, PCR amplification, and sequencing

Total DNA from the microbial communities was extracted using an OMEGA Soil DNA Kit (D5625-01) (Omega Bio-Tek, Norcross, GA, United States) according to the manufacturer’s instructions. The V3-V4 region of the 16S rRNA gene was processed, and PCR was performed with primers (primer-F: ACTCCTACGGGAGGCAGCA and primer-R: GGACTACHVGGGTWTCTAAT). The amplification products were stored at −20°C after amplification. Microbial diversity sequencing of the samples was completed (Personalbio Biotechnology Co., Ltd., Shanghai, China). PCR products were first quantified using the Quant-iT PicoGreen dsDNA Assay Kit, and the samples were then mixed according to the amount of data required for each sample. Library construction was performed using the TruSeq Nano DNA LT Library Prep Kit (Illumina, United States). The qualified libraries were subjected to 2 × 250 bp paired-end sequencing using a NovaSeq 6000 SP Reagent Kit (500 cycles) on the Illumina NovaSeq platform. Problematic samples were retested and subjected to additional preliminary screening. The library and samples were divided, and the barcode sequences were removed according to the index and barcode information. Primer removal, quality filtering, and denoising of sequences were performed according to the DADA2 method (Callahan et al., 2016). Species taxonomy-level annotation and clustering were performed based on sequence alignment. The alpha diversity index in each sample was evaluated based on the distribution of operational taxonomic units (OTUs) in the different samples.

The alpha diversity index and relative abundances of bacteria were calculated using QIIME2 (version 1.8.0) software. The Chao1 index (Chao, 1984) and observed species index characterize microbial richness, while the Shannon and Simpson indices characterize microbial diversity. In addition, the relative abundances of the microbial communities in the different samples were analyzed using QIIME2 (version 1.8.0) and R (version 4.3.1) at the genus and phylum levels. LDA effect size (LEfSe) analysis was performed using the “Python LEfSe” package in R (version 4.3.1).

### 2.5 Statistical analyses

All the data are expressed as the mean ± standard deviation. Excel (version 2019) was used for the data processing of growth indicators, chemical indicators, and fermentation indicators. Multiple comparisons and one-way ANOVA (*p* < 0.05) were performed using SPSS (version 19.0) software to explore the effect of mixed sowing of waxy maize and fodder soybeans on the waxy maize ears, aboveground biomass, chemical compositions, and fermentation quality of silage. The data of the electronic nose odor analysis were calculated using Excel (version 2019) and graphed using Origin (version 2022) and Excel (version 2019). Microbial diversity data were statistically analyzed using R (version 4.3.1). The Shannon, Simpson, and Chao1 indices of the bacterial communities were calculated with the “ggplot2” package. RDA was performed to analyze bacteria and fermentation indicators under different combinations using Canoco (version 5). Mantel tests were used to evaluate the relationships between plant mixed sowing, the chemical and fermentation indicators of silage, and bacterial diversity via the “vegan” package in R (version 4.3.1).

## 3 Results

### 3.1 Effects of different mixed sowing ratios on the waxy maize ears and aboveground biomass

The changes in the ear yield and quality of the waxy maize and the aboveground biomass under the different mixed sowing ratios are shown in Table 1. The percentage of first-grade commercial ears generally increased with increasing proportion of fodder soybeans, reaching a maximum of 64% in the A3. There was no significant difference in the single plant weight of waxy maize straw or fodder soybean between the mixed sowing treatment and CK, but the aboveground biomass generally tended to increase, with A3 and A4 exhibiting the highest weight (0.94 kg), which was 16.00% greater than that of the CK. In general, the weight and quality of the waxy maize ears were not affected by the mixed sowing with different proportions of fodder soybeans.

**Table 1:** Effects of the different mixed sowing ratios on the waxy maize ears and aboveground biomass.

Index	CK	A1	A2	A3	A4
Weight of ears (kg/plant)	0.45 ± 0.05a	0.45 ± 0.04a	0.43 ± 0.08a	0.44 ± 0.02a	0.39 ± 0.06a
Number of maize ears (per plant)	1.00 ± 0.01a	1.00 ± 0.01a	1.04 ± 0.04a	1.04 ± 0.04a	1.00 ± 0.01a
The number of first-grade commercial ears (per plant)	0.54 ± 0.12a	0.58 ± 0.14a	0.62 ± 0.10a	0.67 ± 0.01a	0.58 ± 0.04a
The percentage of first-grade commercial ears (%)	54	58	60	64	58
Fresh weight of waxy maize straw (kg/plant)	0.81 ± 0.50a	0.76 ± 0.80a	0.82 ± 0.80a	0.71 ± 0.50a	0.71 ± 0.70a
Fresh weight of fodder soybeans (kg/plant)	-	0.06 ± 0.10a	0.05 ± 0.10a	0.07 ± 0.10a	0.06 ± 0.10a
Aboveground biomass (kg)	0.81 ± 0.10a	0.83 ± 0.10a	0.93 ± 0.20a	0.94 ± 0.10a	0.94 ± 0.10a

The fresh weight of the fodder soybeans in the table is the average weight of each plant in different proportions. Aboveground biomass is the sum weight of the waxy maize straw and fodder soybeans after the ears were harvested. Different letters indicate significant differences in each line (*p* < 0.05).

### 3.2 Sensory evaluation and electronic nose odor analysis of the silage of different combinations

Silage was evaluated according to the criteria of sensory evaluation (Supplementary Table 1). All the combinations were free of butyric acid and musty odors, with an obvious aromatic flavor and complete stem and leaf structures, and the silage grade was excellent. The treatments were light brown except for the A3, which was light yellow (Supplementary Table 2).

**Figure 1:**
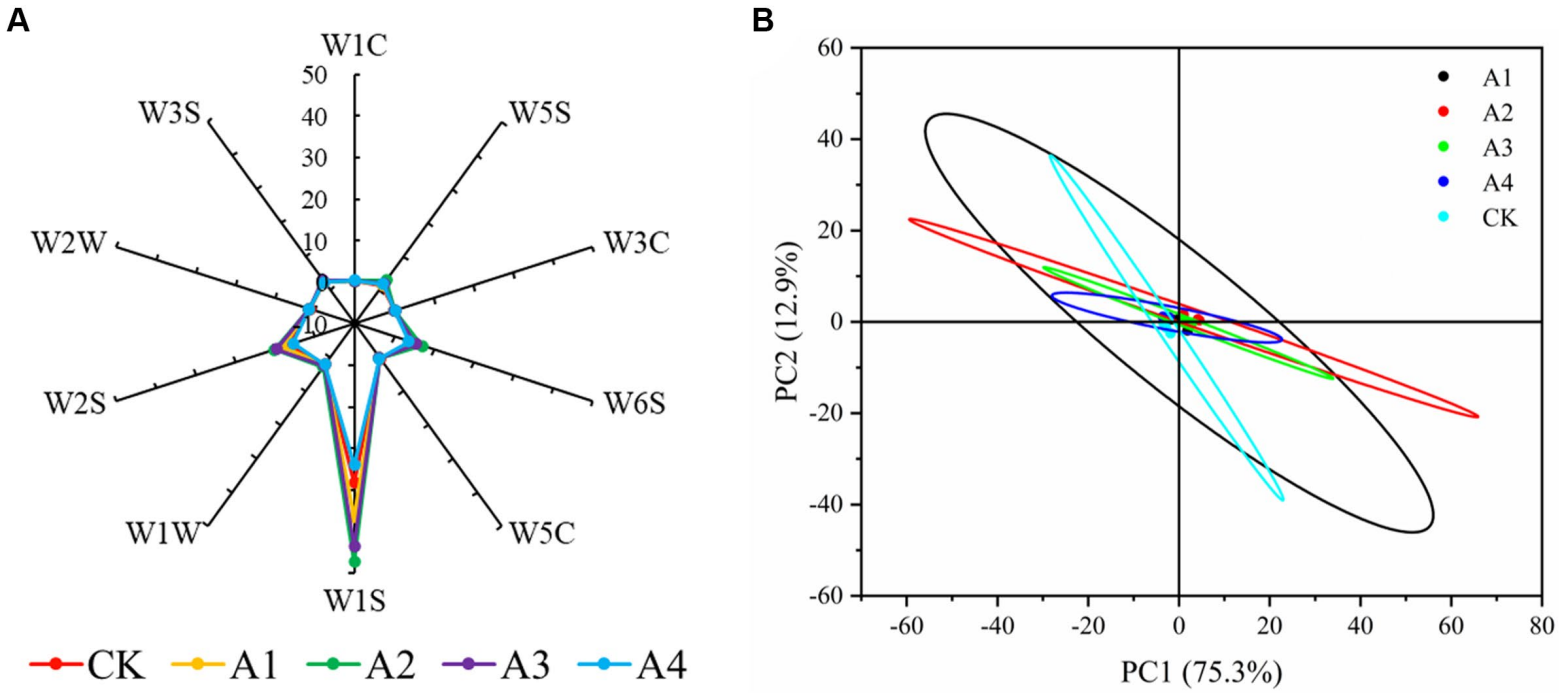
Electronic nose odor analysis **(A)** and PCA **(B)** in the silage samples of the different combinations.

An electronic nose can be used to detect fungal contamination and the presence of fungimycin, which can be used to monitor feed safety (Karakaya et al., 2020). An electronic nose analysis showed that the W1S, W2S, and W6S response values fluctuated slightly among the treatments, and there was no significant difference in the response values of the other categories (Figure 1A, Supplementary Table 3), indicating that the contents of alkanes, alcohols, aldehydes, ketones, and hydrides in the different treatments changed only slightly, and the content of alkanes was relatively high. Principal component analysis (PCA) revealed that the variance contribution rates of PC1 and PC2 were 75.3% and 12.9%, respectively, and the cumulative variance contribution rate was 88.2%, which could represent the main odor value of the silage volatility (Figure 1B). The overall odor of all the treatments was similar, indicating the good quality of the silage.

### 3.3 Chemical component analysis in the silage of different combinations

The chemical compositions of the different silages are shown (Table 2). The DM content significantly differed between the treatments and the CK, and that of A2 was the highest (224.70 g/kg FM). The CP content in all the treatments was greater than that in CK, and the CP content of A3 reached a maximum value of 111.70 g/kg DM, which was 2.29% greater than that in CK. The EE content showed a significant decreasing trend when the ratio was 1:3, but increased in A4. The contents of ADF and NDF decreased gradually with increasing proportions of fodder soybean. When the waxy maize to fodder soybeans ratio was greater than 1:1, the ADF and NDF contents of all the treatments were significantly lower than those of the CK. Compared with that in the CK, the WSC content in the treatments decreased significantly, and the lowest WSC content was 7.62 g/kg DM in A3. There was no significant difference in Ash content between the treatments and the CK except for A1. With the increasing proportion of mixed sowing of fodder soybeans, the RFV of the silages increased gradually.

**Table 2:** Chemical component analysis in the silage of different combinations.

Silage	DM (g/kg FM)	CP (g/kg DM)	EE (g/kg DM)	ADF (g/kg DM)	NDF (g/kg DM)	WSC (g/kg DM)	Ash (g/kg DM)	RFV
CK	204.70 ± 2.00b	88.80 ± 7.00b	62.13 ± 6.17a	467.03 ± 10.41a	557.53 ± 11.66a	33.30 ± 7.48a	85.63 ± 2.48ab	87.63
A1	186.30 ± 2.00c	99.90 ± 4.00ab	52.76 ± 5.45b	458.32 ± 43.61ab	535.75 ± 22.18ab	16.48 ± 1.74b	91.20 ± 3.57a	92.36
A2	224.70 ± 1.00a	92.00 ± 3.00b	47.81 ± 8.82bc	426.44 ± 9.47abc	507.12 ± 22.89abc	13.54 ± 6.59b	75.98 ± 6.49b	102.15
A3	188.00 ± 2.00c	111.70 ± 7.00a	40.78 ± 3.50c	403.72 ± 30.19bc	493.57 ± 28.05bc	7.62 ± 0.97b	71.16 ± 19.29b	108.27
A4	215.00 ± 1.00ab	105.2 ± 7.00ab	64.48 ± 4.71a	398.42 ± 7.45c	462.83 ± 26.81c	10.81 ± 2.03b	77.75 ± 2.14b	116.31

FM, Fresh matter; DM, Dry matter; CP, Crude protein; EE, Crude fat; ADF, Acid detergent fiber; NDF, Neutral detergent fiber; WSC, Water-soluble carbohydrate; Ash, Crude ash; and RFV, Relative feeding value. Different letters indicate significant differences in each column (*p* < 0.05).

### 3.4 Fermentation quality analysis in the silage of different combinations

The fermentation quality of all combinations after 45 days silage is shown in Table 3. The pH of A4 was the highest (4.49) and was significantly greater than that of the CK. The AN concentration changed significantly among all the treatments, and the AN concentration of A3 and A4 was significantly different from that of CK. The AN concentration in A3 was the lowest, reaching 57.20 g/kg TN. With increasing proportions of mixed sowing of fodder soybeans, the LA content in all the treatments significantly differed from that in the CK and showed a decreasing trend, and that in the CK exhibited the greatest difference. Furthermore, the AA content decreased gradually in the treatments, but that in the CK was the lowest. The PA content of each treatment group significantly decreased compared with that of the CK. The BA content was not detected, indicating that no deterioration occurred in any of the silages, consistent with the sensory evaluation.

**Table 3:** Fermentation quality analysis of the silage samples under the different mixed combinations.

Silage	pH	AN (g/kg TN)	LA (g/kg DW)	AA (g/kg DW)	PA (g/kg DW)	BA (g/kg DW)
CK	3.89 ± 0.01c	77.20 ± 7.00bc	8.61 ± 0.01a	0.42 ± 0.01e	0.020 ± 0.00a	ND
A1	3.90 ± 0.01c	94.60 ± 7.00ab	6.64 ± 0.01b	0.80 ± 0.01a	0.017 ± 0.00b	ND
A2	4.15 ± 0.01b	72.50 ± 4.00c	5.45 ± 0.01c	0.72 ± 0.03b	0.017 ± 0.00b	ND
A3	4.08 ± 0.01bc	57.20 ± 4.00d	6.72 ± 0.01b	0.58 ± 0.03c	0.017 ± 0.00b	ND
A4	4.49 ± 0.01a	116.50 ± 9.00a	4.02 ± 0.01d	0.50 ± 0.00d	0.017 ± 0.00b	ND

DM, Dry matter; AN, Ammonia nitrogen; TN, Total nitrogen; LA, Lactic acid; AA, Acetic acid; PA, Propionic acid; BA, Butyric acid; ND, Not detected. Different letters indicate significant differences in each column (*p* < 0.05).

### 3.5 Microbial communities in the silage of different combinations

After microbial sequencing was performed on the silage samples, 1,361,659 raw reads were obtained. The results of the alpha diversity analysis of the mixed silages are shown in Table 4. The coverage of all samples was greater than 0.98, indicating that most of the microbial community composition was fully covered by the sequencing results. The Chao1 index of CK was the highest, while that of A3 was the lowest, indicating that it had the lowest microbial richness. The Shannon index and Simpson index of CK were both greater than those of A1 to A4, and the minimum Shannon and Simpson indices were detected in A2, which indicated that it had the lowest species diversity.

**Table 4:** Alpha diversity analysis of the silage samples from the different combinations.

Silage	Raw reads	Observed species	Chao1 index	Simpson index	Shannon index	Goods coverage
CK	122,196	1746	2195.44	0.95	7.21	0.9835
A1	72,119	470	496.38	0.91	4.87	0.9981
A2	86,416	222	269.87	0.77	3.58	0.9981
A3	54,048	204	227.55	0.83	3.80	0.9989
A4	69,333	296	328.33	0.83	3.94	0.9983

**Figure 2:**
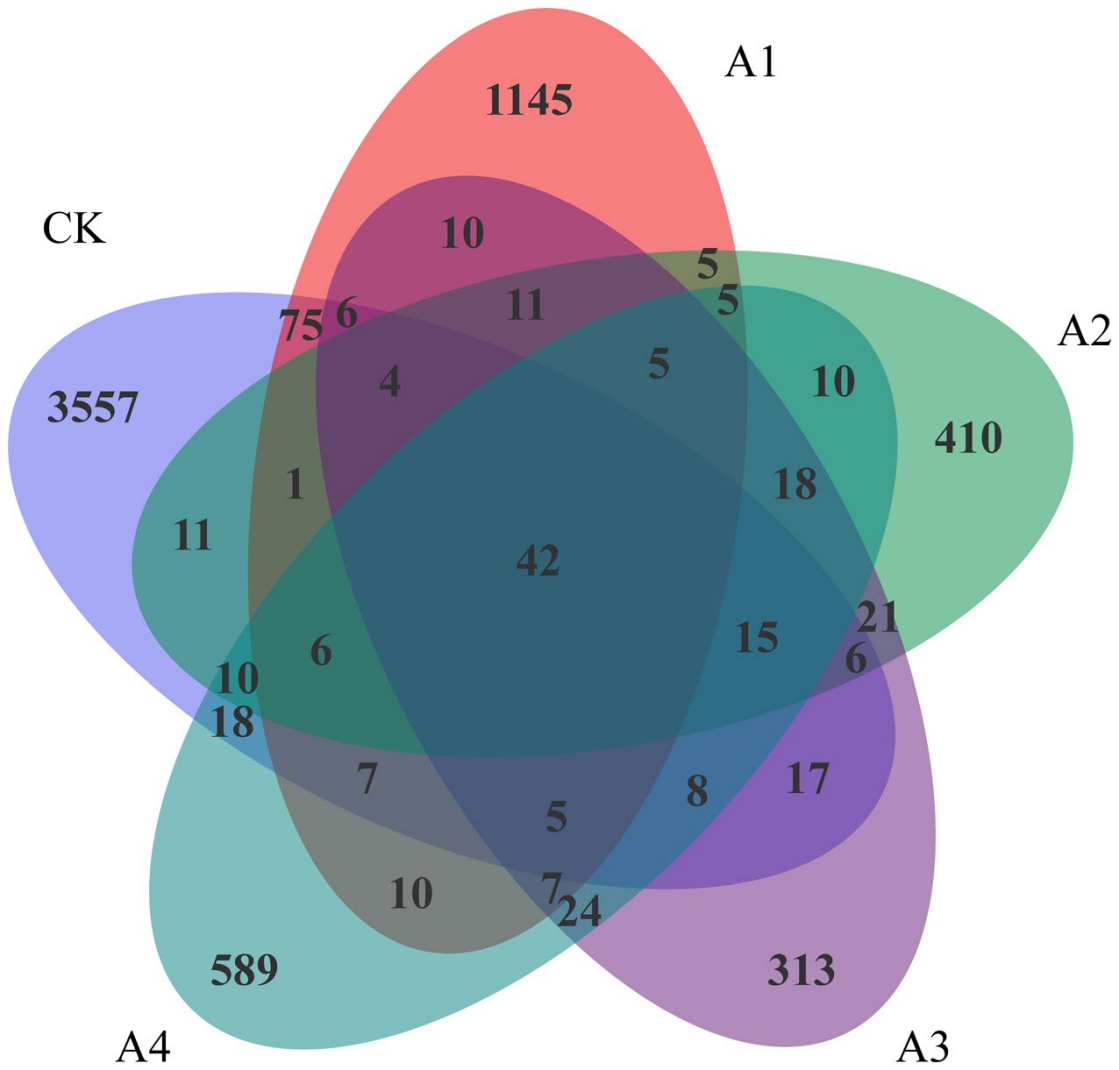
Venn analysis of the operational taxonomic units (OTUs) in the silage samples from the different combinations.

There were 42 overlapping OTUs among the microbial communities of the five samples (Figure 2). There were 3,557 unique OTUs in CK. With the increasing ratio of waxy maize to fodder soybeans from 1:1 to 1:3, the number of unique OTUs in the mixed silage treatments decreased significantly, and the number of unique OTUs in A3 was the lowest, accounting for 313 species. The number of OTUs in the A2 and A4 treatments was slightly greater than that in the A3treatment. Compared with those in the treatments with LDA scores greater than 4.0, *Betaproteobacteriales*, *Anaerolineae*, *Ignavibacteria*, *PHOS-HE36*, and *Ignavibacteriales* were significantly more abundant in CK than in the other treatments (Supplementary Figure 1).

**Figure 3:**
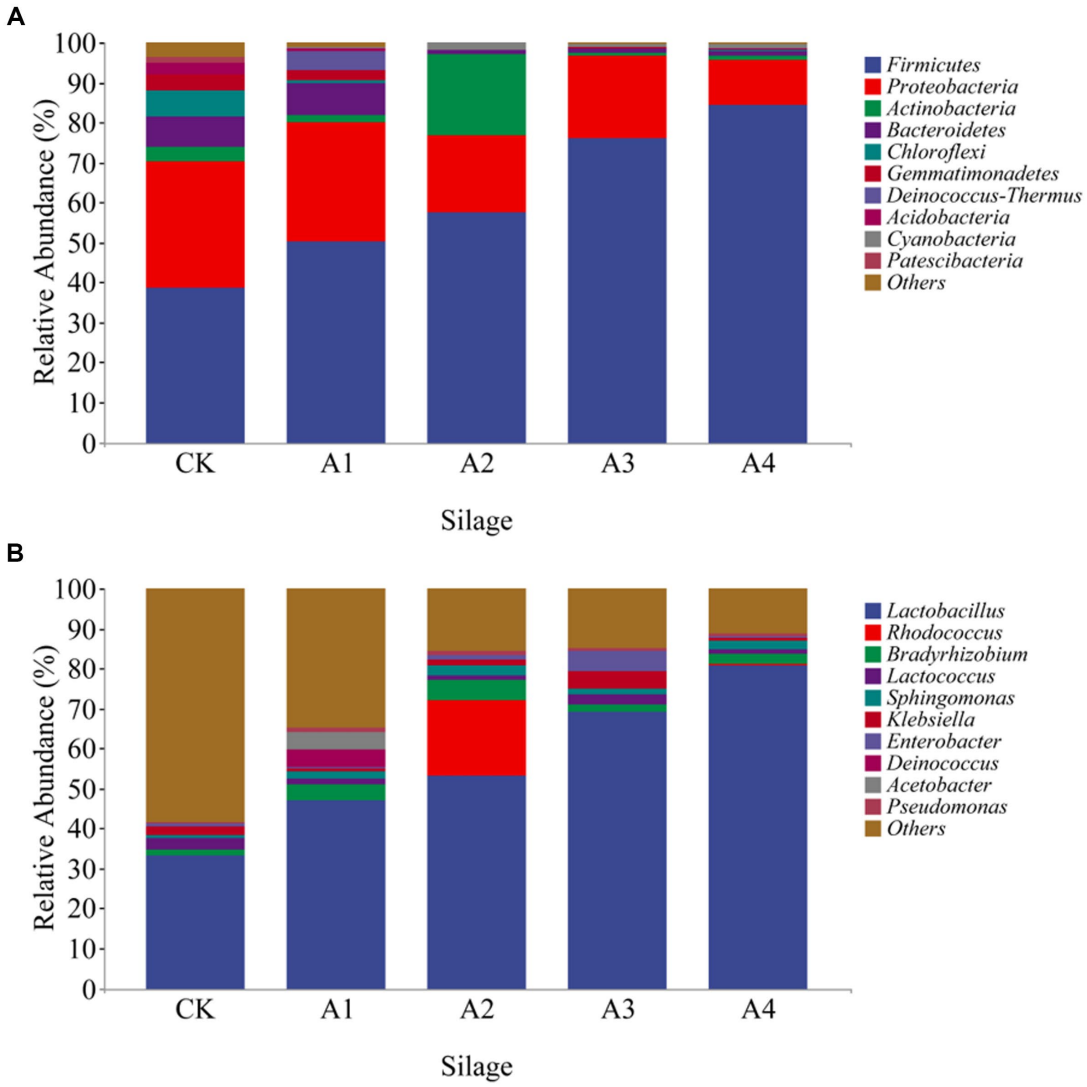
The relative abundance of bacteria at the phylum **(A)** and genus **(B)** levels of the silage samples from the different combinations.

The microbial communities of each treatment were identified, and the relative abundances of the top 10 dominant bacteria in the silage samples at the phylum and genus levels are shown (Figure 3). The microbial communities of all the samples were mainly dominated by *Firmicutes* and *Proteobacteria*. The increase in the abundance of *Firmicutes* was greater than 50% in all treatments with the increase in mixed sowing of fodder soybeans, and that in A4 was the greatest. The relative abundance of *Actinobacteria* in the A2 was 20.36%, which was significantly greater than that in the other treatments. The relative abundance of *Bacteroidetes* was greater in the CK and A1 treatments, at 7.58 and 8.03%, respectively. The relative abundance of *Chloroflexi* in the CK (6.60%) was significantly greater than that in the other treatments. *Lactobacillus* was the dominant bacteria in each silage sample. The relative abundance of *Lactobacillus* was the lowest in CK and increased with increasing of fodder soybean ratios. The relative abundance of *Lactobacillus* in A4 was the highest (80.83%). The relative abundances of *Rhodococcus* (18.93%) and *Bradyrhizobium* (4.83%) were greatest in A2. The relative abundance of *Bradyrhizobium* (1.56%) was lower in the CK than in the other treatments. The relative abundance of *Alloiococcus* (4.50%) in A1 was significantly greater than that in the other treatments (Figure 3B).

### 3.6 Redundancy analysis and Mantel tests under the different combinations

**Figure 4:**
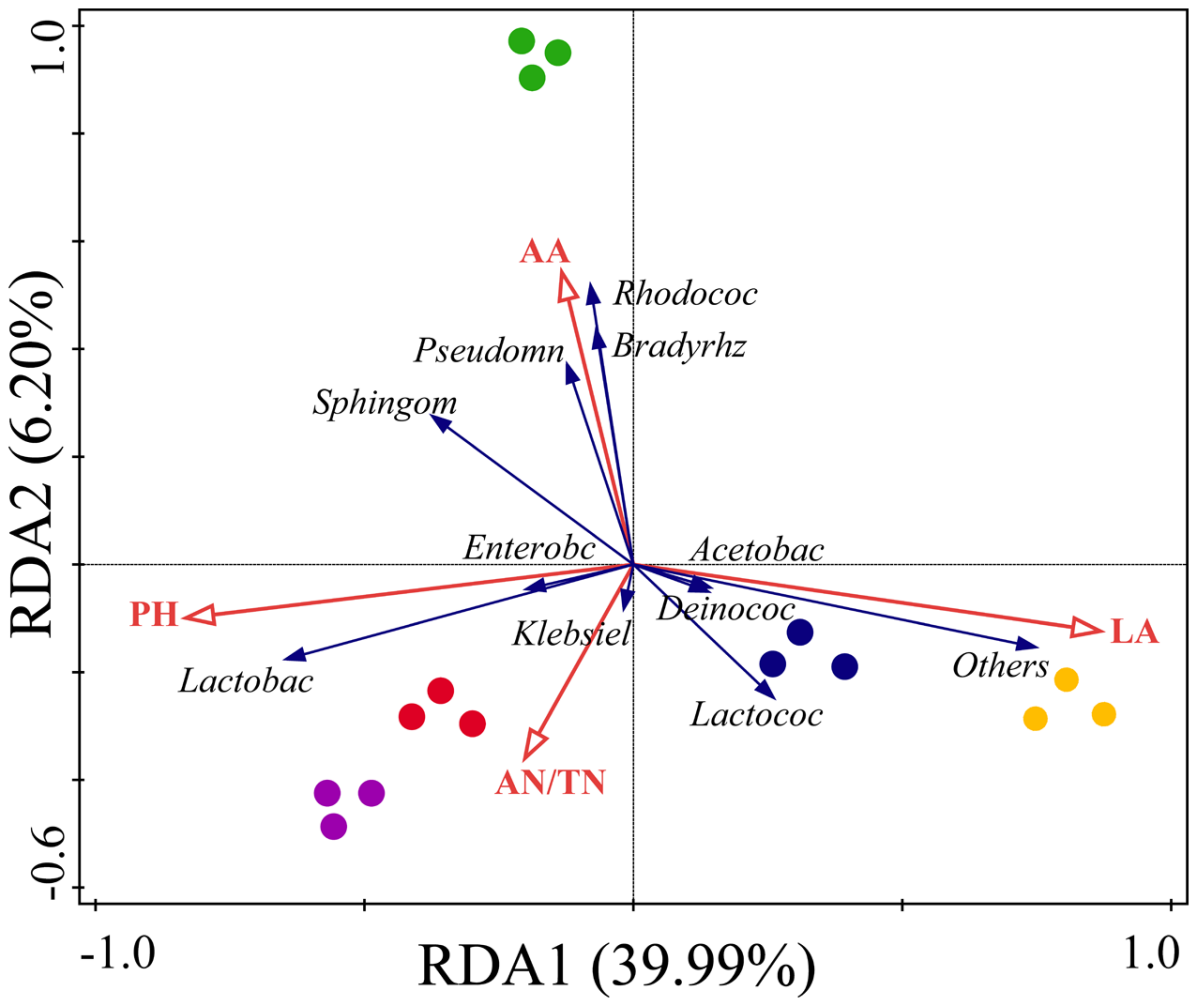
Redundancy analysis (RDA) of the relationship between the bacterial relative abundance and fermentation quality in silage of different combinations. Yellow circle: CK, blue circle: A1, green circle: A2, red circle: A3, purple circle: A4. Red arrows represent fermentation indicators, and blue arrows represent bacteria.

Redundancy analysis revealed that fermentation indicators played a key role and explained 46.19% of the microbial community components (Figure 4). Our results indicate that the fermentation indices affecting the microbial community composition in the silage are inconsistent among the different mixed sowing combinations. The LA content had a greater correlation with the microbial composition of CK and A1, and the AA content had a greater correlation with the microbial composition of A2. Moreover, the concentration of pH and AN was more strongly correlated with the microbial composition of A3 and A4. Meanwhile, pH and LA content had opposite effects on the microbial community composition, while AA content was the fermentation index with the strongest effect on the microbial community composition.

**Figure 5:**
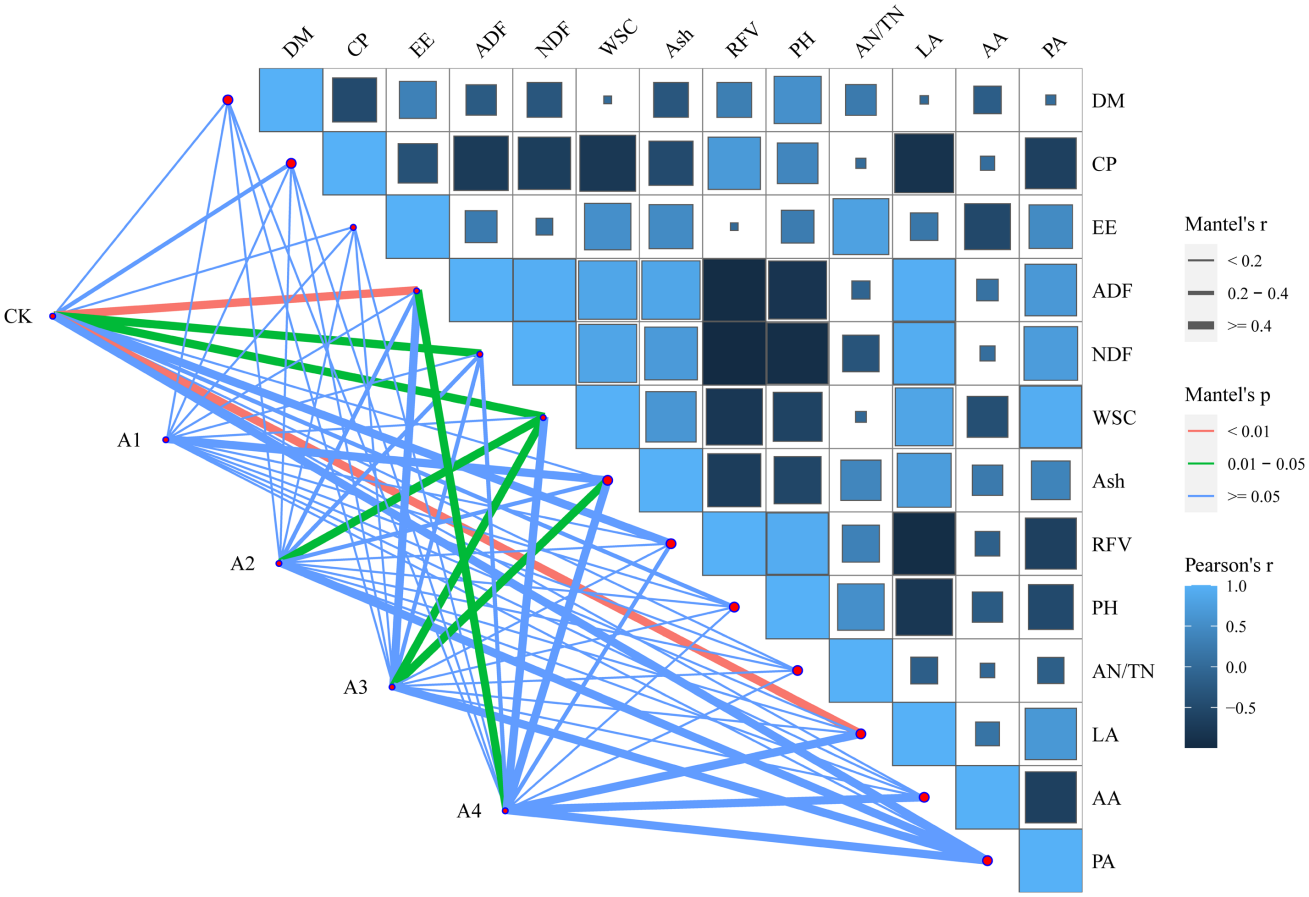
Mantel test analysis of indices and bacterial genera in silage of different combinations.

Mantel tests revealed that the correlation of the indicators with the treatments was inconsistent among the different mixed sowing combinations (Figure 5). ADF, NDF, WSC, and LA contents were significantly related to CK, but no indicator was significantly related to A1. A2 had a significant correlation with only the WSC content, and A3 was significantly related to the WSC and Ash contents. The ADF content was significantly related to A4.

## 4 Discussion

### 4.1 Effects of the different mixed sowing ratios on the yield of waxy maize ears and aboveground biomass

The mixed planting of leguminous and gramineous forages can maximize their morphological and physiological characteristics; fully utilize environmental resources such as water, fertilizer, air, heat, and light during the whole growing season to improve the yield of forages; reduce competition for mineral and nutrient elements in the soil; and achieve complementary utilization (Ledgard et al., 1987; Finn et al., 2013; Tian et al., 2021; Zhang et al., 2023). Studies have shown that the mixed planting of silage maize and fodder soybeans can increase the height and stem diameter of silage maize (Zhan et al., 2013), but the sowing amount has little effect on the plant height and the location of the ears when maize was sown alone (He et al., 2019). In this study, the yield and quality of the waxy maize ears were significantly different between monocultured and mixed sowing plants, indicating that the increasing the proportion of fodder soybeans did not result in a reduction of waxy maize ears. The percentage of first-grade commercial ears in A3 was the greatest, and A3 may be the best mixture of waxy maize and fodder soybeans. Although the yields of the waxy maize stems and fodder soybeans did not change significantly, the aboveground biomass after ears harvesting increased with increasing proportions of fodder soybeans, which was similar to the results of Jiang et al. (2018).

### 4.2 Effects of the different mixed sowing ratios on the chemical components of silages

Chemical components such as WSC and CP are the main factors affecting the fermentation quality and can be used as important indices for evaluating silage quality. WSC acts as a microbial metabolic substrate during the whole fermentation process, and *Lactococcus* species produce LA by decomposing WSC, which reduces the pH of silage and thus prolongs the preservation time of the feed (Xu et al., 2009; Zheng et al., 2011; Bohn et al., 2017). When the waxy maize without ears was used as a single silage, the high WSC content could provide a sufficient substrate for the silage process and ensure fermentation, but the nutrients were insufficient due to the low CP content. However, the addition of mixed sowing of fodder soybeans significantly increased the CP content, which is consistent with the findings of a previous study in which mixed silage of *Melilotus albus* and oat was used (Gu et al., 2011b). Reportedly, ruminant feed requires a CP content of more than 70 g/kg DM to ensure normal ruminal microbial activity, and a low CP content may reduce ruminal microbial proliferation (Silva et al., 2012). In this study, the treatments with increasing CP content benefited from increasing the proportion of mixed sowing of fodder soybeans. The acceptability of feed and animal intake decreases with increasing NDF content, while the digestibility of feed decreases with increasing ADF content (Li et al., 2019). In this study, mixed silage of both waxy maize and fodder soybeans showed significant decreases in the NDF and ADF contents, indicating that mixed sowing effectively improved the feeding quality of the mixed silage, which was consistent with the results of Liu et al. (2017).

### 4.3 Effects of the different mixed sowing ratios on fermentation quality of the silages

The fermentation quality of silage can be significantly affected by the pH and organic acid content, and the feed can remain in a stable state at lower pH. Because of competition for limited nutrients, the relative abundance of LA bacteria increases as the number of harmful bacteria decreases, leading to the production of large amounts of LA, a rapid decrease in pH, and an improvement in the fermentation quality of mixed silage (Li et al., 2020). In this study, the pH of the samples at mixed sowing ratios of 1:1, 1:2, and 1:3 was less than 4.2, possibly because the high WSC content of the waxy maize is suitable for LA production, resulting in better silage quality. However, the pH increased to approximately 4.49 at a mix ratio of 1:4, which may be due to the high proportion of fodder soybeans mixed sowing increasing the buffering capacity. Additionally, a high level of soluble protein can be quickly degraded and form NH^4+^, inhibiting a decrease in pH (Zhang et al., 2017). The main organic acids in silage are LA, AA, PA, and BA, of which LA is a beneficial acid; the amount of LA largely determines the quality of silage. The attached live bacteria can improve the fermentation rate of silage and increase the LA content. With increasing the proportions of mixed sowing of fodder soybeans, the CP content increased, and the WSC content decreased in the treatments, leading to the replacement of the fermentation of isotype LA bacteria by heterotypic LA bacteria (Mu et al., 2020), resulting in a lower LA content in all the treatments relative to the CK, which may also be the reason for the greater AA content in the treatments. In addition, there is a protective mechanism in which fermentation products may convert to weakly acidic compounds in acidic environments (Oude Elferink et al., 2001). Therefore, a portion of the products may be converted to AA because the acidity of AA (PKa = 4.8) is weaker than that of LA (PKa = 3.9). Moreover, an increase in the AA content can also improve the preservation performance of silage, thus delaying secondary fermentation and prolonging the storage time of silage (Gu et al., 2011a). High-quality silage should have a high LA content and a low PA content because AA and PA can more strongly inhibit yeast growth more than LA (Valdez et al., 1988). In this study, the PA content was significantly lower in all the treatments compared with the CK; thus, increasing the proportion of mixed sowing of fodder soybeans improved the aerobic stability of the silage mixture (Oude Elferink et al., 2001). The AN concentration reflects the decomposition of protein and amino acids (Pahlow et al., 2003), and the lowest AN concentration was found in A3, which might indicate decreased protein breakdown. Based on the fermentation quality analysis, the appropriate ratio of waxy maize to fodder soybeans may be 1:3.

### 4.4 Effects of the different mixed sowing ratios on the bacterial diversity of silage

Silage fermentation is a dynamic process involving multiple bacteria and interactions among different microbial communities. The evaluation of silage quality is generally based on changes in fermentation quality and microbial composition as bacteria vary during different fermentation stages (Weinberg et al., 1993). In this study, the abundance and structure of the microbial communities in the CK differed from those in the other treatments. The Chao1 index of all the treatments decreased compared with that of the CK, and the lowest Chao1 index was presented in A3, which indicated that it might have the lowest microbial richness. It has been reported that the diversity of the microbial community decreases with the increasing relative abundance of dominant bacteria (Ogunade et al., 2018). The lowest Shannon and Simpson indices in A2 represented the lowest species diversity. This pattern was similar to that of the OTUs, which gradually decreased with the addition of fodder soybeans. The number of overlapping OTUs of the bacterial communities in all the silages was 42 (Figure 2), which suggested that similar microbial communities coexisted in the silage process of various treatments.

Differences in microbial communities may be a key factor contributing to differences in silage quality (He et al., 2020). A total of four phyla and six genera of microbial flora with relative abundances greater than 1% were detected in the present study, with *Firmicutes*, *Proteobacteria*, *Actinobacteria*, and *Bacteroidetes* as the dominant phyla. As the proportion of mixed sowing of fodder soybeans increased, the abundance of *Firmicutes* gradually increased. The phylum *Firmicutes* ferments sugar into acid and inhibits *Proteobacteria* under anaerobic silage conditions, resulting in a decrease in its abundance (Xin et al., 2023). The dominant genera were *Lactobacillus*, *Bradyrhizobium*, *Lactococcus*, *Sphingomonas*, *Klebsiella*, and *Pseudomonas*. *Lactobacillus*, which plays a dominant role in the anaerobic silage process, utilizes the WSCs, and produces organic acids such as LA and AA, lowering the pH of silage and inhibiting the growth and reproduction of harmful bacteria such as *Clostridium* and *Enterobacteriaceae*. Furthermore, aerobic bacteria such as yeast and molds tend to become dormant, reducing the loss of nutrients and preserving the feed for a long time (Hu et al., 2021). Diversity analysis revealed that various types of bacteria play a role in regulating nutritional components and fermentation quality (Amato et al., 2013), which is the reason why the diversity of microbial communities decreased after silage mixing. In this study, the relative abundances of *Firmicutes* and *Lactobacillus* increased with increasing proportions of mixed sowing of fodder soybeans (Figure 3), suggesting that mixed silage can enhance fermentation quality by changing the structure of bacterial communities. This is similar to the findings of Wang et al., who reported that mixed silage of corn straw and soybean curd residue decreased bacterial diversity and increased the relative abundance of beneficial bacteria (Wang et al., 2022).

### 4.5 Correlation analysis of the silage quality and the microbial community

The abundances of some genera are strongly correlated with both nutritional and fermentation quality. The significant separation of the bacterial communities in the mixed silage from the different treatments (Figure 4) indicated that increasing the proportion of mixed sowing of fodder soybeans influenced the composition of the microbial community. The microbial community of the silage was more affected by the ingredient ratio in A3 and A4 and was less affected in A1. In this study, *Lactococcus* had a positive correlation with LA content, and *Deinococcus* and *Acetobacter* also had some correlation with LA content. It has been shown that *Acetobacter* is a specialized aerobic bacterium capable of partially oxidizing alcohol or sugar to organic acids, which may help inhibit fungal colonization and prolong the aerobic stability of silage (Xian et al., 2022). The reported prevalence of *Acetobacter* in silage is very controversial, and in the present study, only A1 had a relative abundance of Acetobacter (4.28%) greater than 1%. *Lactococcus*, a parthenogenetic anaerobic bacterium, is normally the most abundant in the early stages of silage when the growth and multiplication of *Lactococcus* are inhibited during the anaerobic process (Zeng et al., 2020). However, *Acetobacter* may promote the growth of *Lactococcus* (1.20–2.65%) and cause the proliferation of *Sphingomonas*, and the correlation between pH and *Sphingomonas* in the RDA was similar to that reported by Xin et al. (2023). However, the abundance of *Lactobacillus* still significantly increased with the addition of fodder soybeans. Studies have shown that *Lactobacillus* can produce β-glucosidase, degrade cellobiose, and promote the fermentation of LA bacteria to produce LA (Qiao et al., 2013), which is consistent with the results showing that the pH determined by RDA was significantly correlated with *Lactobacillus*. *Sphingomonas*, *Rhodococcus*, *Bradyrhizobium,* and *Pseudomonas* had a positive effect on AA (Figure 4), suggesting that these bacteria can increase the AA content, which is similar to the previous reports (Guan et al., 2018). The Mantel test showed that the composition of the bacterial community in the treatments with different proportions of fodder soybeans was significantly affected by the contents of ADF, WSC, and Ash (Figure 5), and the contents of ADF and WSC gradually decreased after ensiling (Tables 2, 3), which might be the result of the increase in the abundance of *Firmicutes* and the decrease in the abundance of *Proteobacteria* in the fermentation environment (Figure 3) (Qiao et al., 2013). The Mantel test and RDA both showed a high correlation between CK and LA, which may be due to the high WSC content resulting in the production of large amounts of LA. The correlation between A3 and WSC may be due to the significant decrease in the WSC content with the addition of fodder soybeans, and the change in feedstuffs leading to a significant change in the microbial community of the silage, which echoes the apparent separation of A3 from CK in the RDA. The combined analysis suggested that mixing waxy maize with fodder soybeans can improve silage quality by changing the microbial environment of the silage.

In conclusion, the mixed sowing of waxy maize and fodder soybeans in different proportions had no effect on the yield and quality of the waxy maize ears. Compared with waxy maize straw silage alone, mixed silage of waxy maize and fodder soybeans after ear harvesting had an altered microbial community structure, increased relative abundances of beneficial bacteria, decreased relative abundances of harmful bacteria, and improved nutritional and fermentation quality. Comprehensive analysis revealed that the optimal mixed sowing ratio for waxy maize to fodder soybeans was 1:3. This study demonstrated that the mixed sowing of waxy maize to fodder soybeans for mixed silage can improve the utilization value of aboveground biomass and lays a theoretical foundation for improving the utilization of waxy maize straw in animal husbandry.

## Data availability statement

The original contributions presented in the study are included in the article/Supplementary material, further inquiries can be directed to the corresponding authors.

## Author contributions

MY: Conceptualization, Writing – original draft, Writing – review & editing, Data curation, Methodology. FW: Data curation, Writing – review & editing. WX: Data curation, Writing – original draft. XL: Data curation, Writing – original draft. HY: Methodology, Writing – review & editing. MT: Methodology, Writing – original draft. RQ: Methodology, Writing – original draft. BL: Conceptualization, Writing – review & editing. GC: Conceptualization, Writing – review & editing.
