# Supplementary material for: Effects of the fermentation quality and microbial community of waxy maize mixed with fodder soybean silage
Source: Front Microbiol. 2024 May 3;15:1405018. doi: 10.3389/fmicb.2024.1405018 (PMC11099260; doi:10.3389/fmicb.2024.1405018)
Supplement: Supplementary file 1 [file Data_Sheet_1.docx]

Supplementary Material

# Supplementary Figures and Tables

## Supplementary Figures


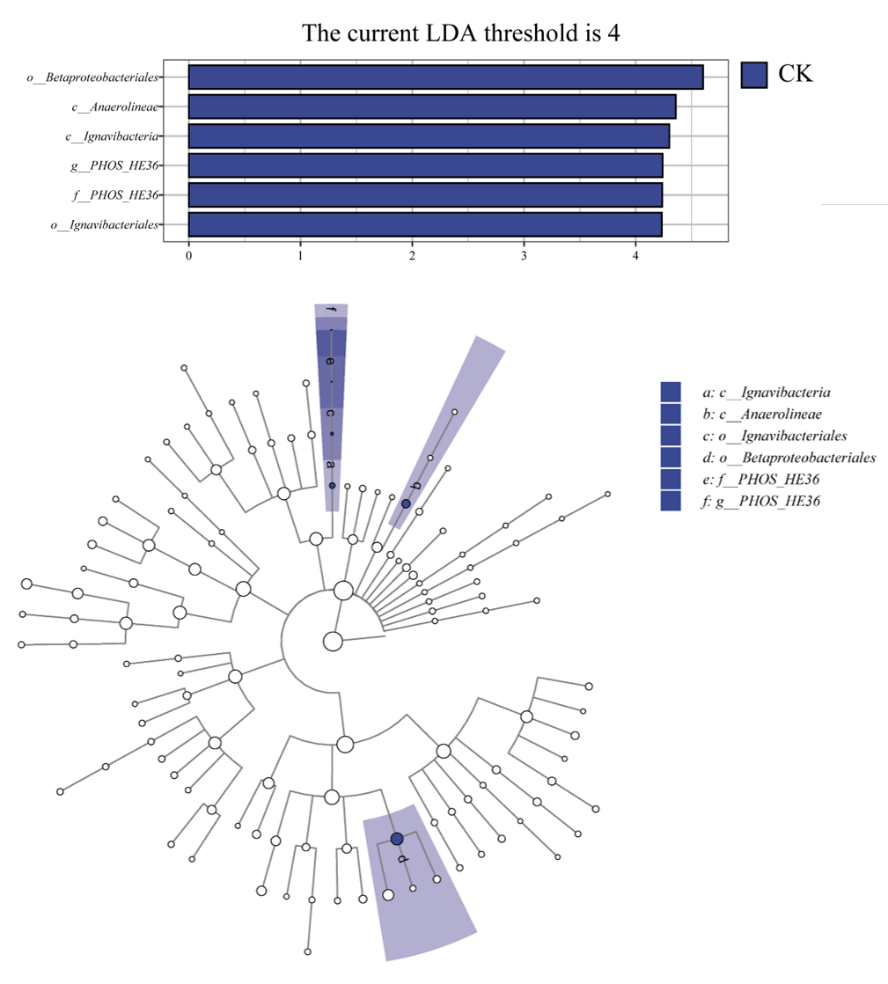


**Supplementary Figure 1.** Comparison of bacterial variations in mixed silage using the LEfSe online tool.

## Supplementary Tables

**Supplementary Table 1.** Sensory evaluation criteria of silage.

| Item | Evaluation Standards | | | Score |
| --- | --- | --- | --- | --- |
| Smell | There was no butyric acid odor, and was a fragrant fruity or distinct bread aroma in the sample. | | | 14 |
|  | There was a weak butyric acid odor, a strong sour odor, and a weak aromatic odor in the sample. | | | 10 |
|  | The taste of butyric acid was quite strong in the sample, or there was a pungent burnt odor or musty odor. | | | 4 |
|  | There was a strong butyric acid or ammonia odor, or almost no acid odor in the samples. | | | 2 |
| Texture | The stem and leaf structure were well maintained. | | | 4 |
|  | The leaf structure was poorly maintained. | | | 2 |
|  | The stem and leaf structure were poorly maintained or mildly contaminated with mold. | | | 1 |
|  | Stems and leaves were rotten or seriously polluted. | | | 0 |
| Color | Similar to the raw material, it was light brown after drying. | | | 2 |
|  | Slightly discolored, it was yellowish or brownish. | | | 1 |
|  | Discoloration is severe, it was dark green or faded yellow, with a strong musty smell. | | | 0 |
| Total score | 16-20 | 10-15 | 5-9 | 0-4 |
| Grade | Excellent | Good | Medium | Rotten |

**Supplementary Table 2.** Sensory evaluation of different storage.

| Treatment | Color | Smell | Texture | Total score | Grade |
| --- | --- | --- | --- | --- | --- |
| CK | 2 | 12 | 3 | 17 | Excellent |
| A1 | 2 | 12 | 2 | 16 | Excellent |
| A2 | 2 | 13 | 2 | 17 | Excellent |
| A3 | 1 | 13 | 3 | 17 | Excellent |
| A4 | 2 | 13 | 2 | 17 | Excellent |

**Supplementary Table 3.** Sensor arrays and their performance description.

| Name | Performance description |
| --- | --- |
| W1C | Sensitive to aromatic compounds |
| W5S | Sensitive to nitrogen oxides |
| W3C | Sensitive to nitrogen and aromatic compounds |
| W6S | Sensitive to hydrogenated substances |
| W5C | Sensitive to aromatic olefins and polar compounds |
| W1S | Sensitive to alkane compounds |
| W1W | Sensitive to sulfurized substances |
| W2S | Sensitive to alcohols and aldehydes |
| W2W | Sensitive to sulfur-containing organics and aromatic components |
| W3S | Sensitive to long chain alkanes |
